# Reduced penetrance of MODY-associated *HNF1A/HNF4A* variants but not *GCK* variants in clinically unselected cohorts

**DOI:** 10.1016/j.ajhg.2022.09.014

**Published:** 2022-10-17

**Authors:** Uyenlinh L Mirshahi, Kevin Colclough, Caroline F Wright, Andrew R Wood, Robin N Beaumont, Jessica Tyrrell, Thomas W Laver, Richard Stahl, Alicia Golden, Jessica M Goehringer, Geisinger-Regeneron DiscovEHR Collaboration, Timothy F Frayling, Andrew T Hattersley, David J Carey, Michael N Weedon, Kashyap A Patel

**Affiliations:** 1 Geisinger Clinic, Geisinger Health System, Danville, PA, USA; 2 Molecular Genetics, Royal Devon and Exeter NHS Foundation Trust, Exeter, UK; 3 Institute of Biomedical and Clinical Science, College of Medicine and Health, University of Exeter, Exeter, UK

**Keywords:** monogenic diabetes, monogenic disease, MODY, HNF1A, HNF4A, GCK, prevalence, penetrance, population screening, ACMG, variant curation

## Abstract

The true prevalence and penetrance of monogenic disease variants are often not known because of clinical-referral ascertainment bias. We comprehensively assess the penetrance and prevalence of pathogenic variants in *HNF1A*, *HNF4A*, and *GCK* that account for >80% of monogenic diabetes. We analyzed clinical and genetic data from 1,742 clinically referred probands, 2,194 family members, clinically unselected individuals from a US health system-based cohort (n = 132,194), and a UK population-based cohort (n = 198,748). We show that one in 1,500 individuals harbor a pathogenic variant in one of these genes. The penetrance of diabetes for *HNF1A* and *HNF4A* pathogenic variants was substantially lower in the clinically unselected individuals compared to clinically referred probands and was dependent on the setting (32% in the population, 49% in the health system cohort, 86% in a family member, and 98% in probands for *HNF1A*). The relative risk of diabetes was similar across the clinically unselected cohorts highlighting the role of environment/other genetic factors. Surprisingly, the penetrance of pathogenic *GCK* variants was similar across all cohorts (89%–97%). We highlight that pathogenic variants in *HNF1A*, *HNF4A*, and *GCK* are not ultra-rare in the population. For *HNF1A* and *HNF4A*, we need to tailor genetic interpretation and counseling based on the setting in which a pathogenic monogenic variant was identified. *GCK* is an exception with near-complete penetrance in all settings. This along with the clinical implication of diagnosis makes it an excellent candidate for the American College of Medical Genetics secondary gene list.

## Teaser

The prevalence of pathogenic variants in common MODY-associated genes are ∼1:1,500 in the population. The penetrance of pathogenic *HNF1A* and *HNF4A* variants, but not of *GCK* variants, is substantially lower when found incidentally. Our findings are important for incidental reporting of pathogenic variants in MODY and other monogenic disorders.

## Introduction

Maturity-onset diabetes of the young (MODY [MIM: 606391]) is the most common subtype of monogenic diabetes. It is an autosomal-dominant form of the monogenic disease and classically presents with diabetes before 25 years of age.^1^^,^^2^ Pathogenic variants in *HNF1A* (MIM: 142410), *HNF4A* (MIM: 600281), and *GCK* (MIM: 138079) account for >80% of all monogenic diabetes.^3^ Pathogenic variants in *HNF1A* and *HNF4A* cause progressive beta-cell dysfunction leading to diabetes whereas pathogenic variants in *GCK* cause stable mild hyperglycemia.^2^^,^^4^ Identification of MODY is clinically important due to its impact on the treatment of diabetes.^2^^,^^4^ *HNF1A*/*HNF4A*-MODY (*HNF1A*-MODY, MIM: 600495; *HNF4A*-MODY, MIM: 125850) are better treated with oral sulphonylureas whereas *GCK*-MODY (MIM: 125851) does not need treatment and is not at high risk of diabetes-related complications.^2^^,^^4^^,^^5^^,^^6^^,^^7^

An accurate estimate of the prevalence and penetrance of diabetes associated with pathogenic variants in MODY-associated genes is needed for genetic counselling, reporting incidental findings, and resource planning. Due to the treatment implications, the American College of Medical Genetics and Genomics (ACMG) recommends genetic laboratories report the pathogenic variants in *HNF1A* when they are identified incidentally.^8^ This can be identified either as part of research or investigation of rare genetic diseases other than MODY. The reducing cost of sequencing allows exome and genome sequencing to become ubiquitous. It is now being used as a first-line test in clinical genetic testing for monogenic diseases and is even available direct to consumers.^9^^,^^10^ There is also a move toward genome sequencing at birth such as the Newborn Genomes Programme in the UK https://www.genomicsengland.co.uk/initiatives/newborns). This paradigm shift means more individuals are identified with pathogenic variants before the onset of disease. However, the crucial part of reporting pathogenic variants particularly identified incidentally is to accurately inform the risk of diabetes. The lack of this information will seriously compromise our ability to counsel individuals and will reduce the benefit of reporting the incidental variants. Identifying the accurate risk and the prevalence of pathogenic variants in clinically selected and clinically unselected settings will generate much-needed evidence to influence the future ACMG secondary gene list.^8^

Our understanding of the risk of diabetes and the prevalence of estimates of pathogenic variants in MODY-associated genes are predominantly based on clinically selected cohorts. It is well recognized that estimates based on clinically selected individuals are likely to be overinflated.^11^ There are some studies conducted in unselected cohorts, but they are limited by sample size (<2,000 to 39,000),^12^^,^^13^ low number of individuals with pathogenic variants (≤5 for *HNF1A*), or studied atypical relatively common variants from array genotyping.^14^ These studies also lacked direct comparison with clinically selected cohorts or family members to comprehensively assess change in disease risk from clinically selected cohorts to clinically unselected cohorts from different settings.

Large-scale research studies such as the UK Biobank (N = 500,000) and health center studies such as the Geisinger DiscovEHR (N = 180,000) have extensive clinical and health record data.^15^^,^^16^^,^^17^ The availability of exome-sequencing data in these studies allows the assessment of the prevalence and penetrance of monogenic disease variants in different settings and with different ascertainment criteria. In this study, we studied these two large clinically unselected cohorts and MODY proband and proband family member cohorts totaling >300,000 individuals to comprehensively assess the prevalence and penetrance of diabetes for the three most common genetic causes of MODY.

## Subjects and methods

### Study populations

#### MODY probands cohort

We included 1,742 probands up to age 85 who were referred to for genetic testing at the Molecular Genomics Laboratory at the Royal Devon and Exeter Hospital, Exeter, UK with a clinical suspicion of MODY from routine primary or specialist clinical care from the UK. They were subsequently found to harbor a pathogenic variant in *HNF1A* (n = 661), *HNF4A* (n = 142), or *GCK* (n = 939). Informed consent was obtained from the probands or their parents/guardians and the study was approved by the North Wales ethics committee. The clinical features of these individuals at referral for genetic testing are shown in Table S1 in the supplemental information.

#### Family members cohort

The family member cohort comprises the individuals up to age 85 who were related (up to a third degree) to the MODY probands (n = 2,194). These individuals were referred from routine clinical care for family genetic screening to the Molecular Genetics Laboratory at the Royal Devon and Exeter Hospital. This included family members for *HNF1A*-MODY probands (n = 954), *HNF4A*-MODY probands (n = 253), and *GCK*-MODY probands (n = 987). Informed consent was obtained from the family members, and the study was approved by the North Wales ethics committee. The clinical features of these individuals are shown in Table S2.

#### Geisinger cohort

The Geisinger cohort is a health-system-based cohort from the USA consisting of 132,194 individuals up to age 85 years who sought healthcare at an outpatient and/or inpatient facility within Geisinger, a health care provider to central and north-eastern Pennsylvania, USA. Individuals consented to participate in the MyCode Community Initiative to create a biorepository of blood, serum, and DNA samples for broad research use, including genomic analysis.^16^ MyCode samples are linked to Geisinger electronic health records (EHR). We used the routinely collected data including clinical diagnosis, procedures, medications, and laboratory results from MyCode participants during their encounters with Geisinger providers in this study. Individuals whose age of diabetes diagnosis could not be determined from their EHR were excluded from the study (n = 2,171/33,415 of all individuals with diabetes). Genetic analysis was carried out as part of the DiscovEHR collaboration between Geisinger and the Regeneron Genetics Center by microarray genotyping and exome sequencing. This study was reviewed by the Geisinger Institutional Review Board and determined as not including human subject research as defined in 45CFR46.102(e) in written consent (Study #2016-0269). Of the total cohort, 87,225 (66%) were unrelated to third-degree relationships. The cohort characteristics at recruitment for exome analysis are summarized in Table S1 and have been described extensively.^17^^,^^18^

#### UK Biobank

UK Biobank is a population-based cohort from the UK with deep phenotyping data and genetic data for around 500,000 individuals aged 40–70 years at recruitment.^15^ Participants provided a range of information via questionnaires and interviews including diabetes status. Additionally, a panel of biomarkers was measured from blood and urine, including random blood glucose and HbA1c.^15^ Phenotypes were derived from medical history interviews, in- and outpatient ICD9 and ICD10 codes, operation codes, and death registry data. A subset of ∼200,000 DNA samples from UK Biobank participants underwent exome sequencing; this dataset was recently made available for research.^19^ This research was conducted using the UK Biobank Resource under UK Biobank project numbers 49847 and 9072. The UK Biobank resource was approved by the UK Biobank Research Ethics Committee and all participants provided written informed consent to participate. Individuals with missing age at diabetes diagnosis were excluded (n = 1,135/12,569 of all individuals with diabetes). Table S1 described the clinical characteristics at recruitment for 198,748 individuals included in the study, of whom 184,142 (93%) were unrelated up to third-degree relationships.

### Definition of diabetes and mild hyperglycemia

Diabetes was defined as (1) self-reported by participants, (2) having an ICD9/10 code for diabetes, (3) being on a diabetes treatment, or (4) having HbA1c ≥ 48 mmol/mol before recruitment.^20^^,^^21^ Mild hyperglycemia was defined accordingly to the American Diabetes Association definition of prediabetes (HbA1c ≥ 39 mmol/mol or fasting glucose ≥ 5.6 mmol/L).^20^ The self-reported lack of diabetes was confirmed using HbA1c or fasting/random glucose in 93.5% of participants in the Geisinger cohort and 95% participants in the UK Biobank.

### Age at diagnosis of diabetes

Age of diabetes diagnosis was self-reported in the UK Biobank at recruitment. For the Geisinger cohort, we used age at the first evidence of diabetes diagnosis from ICD9/10 code or the start of anti-diabetic medication or first HbA1c measurement >48 mmol/mol. Individuals were diagnosed at recruitment based on HbA1c alone. For the individuals who were diagnosed with diabetes by baseline blood test only, the age at recruitment was used as age at diagnosis of diabetes. This applied to 16% (1,833/11,488) of individuals with diabetes in the UK Biobank and 0.7% (213/31,266) of individuals with diabetes in the Geisinger cohort.

### Genetic analysis

#### MODY probands and family members

Sanger sequencing or targeted next-generation sequencing was used to undertake genetic analysis in this study. The detailed method for these assays has been described previously.^22^ Variants in *HNF1A*, *HNF4A*, and *GCK* were analyzed by the clinical scientists at the Molecular Genetics Laboratory at the Royal Devon and Exeter Hospital as part of the routine diagnostic care. This is the only laboratory that provides genetic testing for MODY for the whole UK population. All probands included in this study had variants classified as likely pathogenic (class 4) or pathogenic (class 5). Interpretation and classification of sequence variants were undertaken based on the American College of Medical Genetics and Genomics (ACMG)/Association of Molecular Pathology (AMP) guidelines.^23^ The list of the included variants is shown in Table S3. We annotated variants by clinically used transcripts (GenBank: NM_000545.6 for *HNF1A*, GenBank: NM_175914.4 for *HNF4A*, and GenBank: NM_000162.5 for *GCK*).

#### Geisinger cohort

Individuals underwent exome sequencing as part of the DiscovEHR collaboration of Geisinger (Danville, PA) and the Regeneron Genetics Center (Tarrytown, NY).^17^ The detailed method for exome sequencing has been described previously.^24^ Quality controls included filtering for samples of variants with read depth ≥ 10 (insertions and/or deletions, indels) or ≥ 7 (single-nucleotide variants, SNVs), alternate allele balance >15% for SNVs or >20% for indels, and alternate allele reads >5.

#### UK Biobank

Detailed sequencing methodology for UK Biobank samples is provided by Szustakowski et al.^19^ and is available at https://biobank.ctsu.ox.ac.uk/showcase/label.cgi?id=170. Briefly, exomes were captured with the IDT xGen Exome Research Panel v.1.0 which targeted 39 Mbp of the human genome with coverage exceeding on average 20× on 95.6% of sites. The OQFE protocol was used for mapping and variant calling to the GRCh38 reference. We included variants that had individual and variant missingness <10%, Hardy-Weinberg equilibrium p value >10^−15^, minimum read depth of 7 for SNVs and 10 for indels, and at least one sample per site passed the allele balance threshold > 15% for SNVs and 20% for indels.

#### Variant annotation and classification in UK Biobank and Geisinger cohorts

Variants were annotated by AlaMut batch software v.1.8 (Interactive Biosoftware, France) using clinical transcripts for *HNF1A*, *HNF4A*, and *GCK* as listed above. *HNF1A*, *HNF4A*, and *GCK*-MODY is caused by haploinsufficiency in these genes^25^^,^^26^ and heterozygous pathogenic variants can be missense or protein truncating.^25^^,^^26^ The protein-truncating variants outside the last exon are considered pathogenic for these genes. We defined a protein-truncating variant (PTV) as a variant that is predicted to cause a premature stop-gain or a frameshift or abolish a canonical splice site (−2 or +2 bp from the exon boundary). In this study, we excluded PTVs in the last exon of each gene and only those deemed to be high confidence by the Loss-Of-Function Transcript Effect Estimator (LOFTEE)^27^ were retained. As the disease is caused by the haploinsufficiency in these three genes, the rare protein-truncating variants outside the last exon are considered pathogenic in these genes.^25^^,^^26^^,^^28^

We reviewed all heterozygous missense/PTV variants in UK Biobank and the Geisinger cohort that were observed at minor allele frequency (MAF) < 0.001 in gnomAD v.2 (N = 141,456)^27^ and in each study cohort, respectively. We included variants in the analysis if missense/PTV variants were classified as pathogenic or likely pathogenic based on ACMG/AMP guidelines by clinical scientists at Exeter Molecular Genetic laboratory as part of routine clinical diagnostic care (i.e., previously seen in the MODY probands) and were ultra-rare in the population (maximum allele count of 2 in gnomAD v.2, MAF < 1.4 × 10^−5^). Missense variants that were not seen in MODY probands were classified as either VUSs (variants of uncertain significance) or benign.

Three researchers independently manually reviewed sequence read data for all the pathogenic variants (missense and PTVs) in Integrative Genomics Viewer (IGV)^29^ to remove false-positive variants. The variants considered to be of excellent quality by all three researchers were included in the analysis.

#### Sanger sequencing validation of all pathogenic variants in the Geisinger cohort

We Sanger sequenced 93 samples with pathogenic variants in one of the three genes identified by exome sequencing. Of 93 samples, 29 had the most common *HNF1A* pathogenic variant that is a frameshift variant (GenBank: NM_000545.6 for *HNF1A* c.863_864insC [p.Pro289AlafsTer28]) in exon 4 due to an insertion of a C. This variant is difficult to detect robustly in exome- or genome-sequencing data due to the location in a repetitive poly-C tract and the presence of a common variant at the end of the tract (rs56348580, c.864G>C [p.Gly288Gly], MAF = 0.26). In support of this, the Sanger sequencing confirmed this variant in only 4 of 29 (14%) samples. There were 23 frameshift variants detected in this region in the UK Biobank. Due to high false positive rate, we excluded all *HNF1A* c-insertion variants from the UK Biobank cohort as we were unable to perform Sanger sequencing confirmation. Of the remaining 64 samples in the Geisinger cohort, all were confirmed on Sanger sequencing. However, one sample was mosaic for a pathogenic variant in *HNF4A*, and thus was excluded from the analysis.

### Statistical analysis

Kaplan-Meier survival estimate was used to compute the age-dependent penetrance of diabetes in *HNF1A* and *HNF4A* heterozygotes. Log-rank test for equality was used to compare the penetrance of diabetes between the groups. Cox’s regression was used to compute the hazard ratio for developing diabetes with or without adjustment of covariates. All individuals with pathogenic variants were included in the Kaplan-Meier survival analyses. Fisher’s exact test was used to compare the penetrance of mild hyperglycemia in *GCK* heterozygotes between the cohorts. We used linear regression to compare fasting glucose and HbA1c levels with and without adjustment of covariates between the *GCK* heterozygotes from different cohorts. We used Cochran’s Q test to assess heterogeneity between the study cohorts. All the analysis was performed using Stata 16 (College Station, Texas, USA).

## Results

### Up to 1 in 1,500 individuals in clinically unselected cohort contain a pathogenic variant in one of the MODY-associated genes

In the Geisinger cohort of 132,194 individuals, we observed 14 individuals with pathogenic *HNF1A* variants (prevalence [95% CI] of 0.011% [0.006%–0.018%]), 17 individuals with pathogenic *HNF4A* variants (prevalence of 0.013% [0.008%–0.021%]), and 32 individuals with pathogenic *GCK* variants (prevalence of 0.024% [0.017%–0.0034%], Tables S1, S3, S6, and S10). Similarly, in the UK Biobank cohort of 198,748 individuals, 22 individuals harbor pathogenic *HNF1A* variants (prevalence of 0.011% [0.007%–0.017%]), 29 harbor pathogenic *HNF4A* variants (prevalence of 0.015% [0.010%–0.021%]), and 83 harbor pathogenic *GCK* variants (prevalence of 0.042% [0.034%–0.0053%], Tables S1, S3, S6, and S10). In the aggregate of the three commonest genes for MODY, approximately 1:2,100 (95%CI 1:2,700 to 1:1,640) and 1:1,500 (1:1,750 to 1:1,250) individuals harbor a pathogenic variant for *HNF1A*, *HNF4A*, or *GCK* in the Geisinger and UK Biobank population cohorts, respectively. Using UK Biobank estimate, there are 221,883 and 43,406 individuals with a pathogenic variant for one of these genes in the current US (332.82 M) and UK (65.11 M) populations, respectively.

### Penetrance of pathogenic *HNF1A* variants is lower in clinically unselected cohorts compared to a clinically ascertained cohort

**Figure 1:**
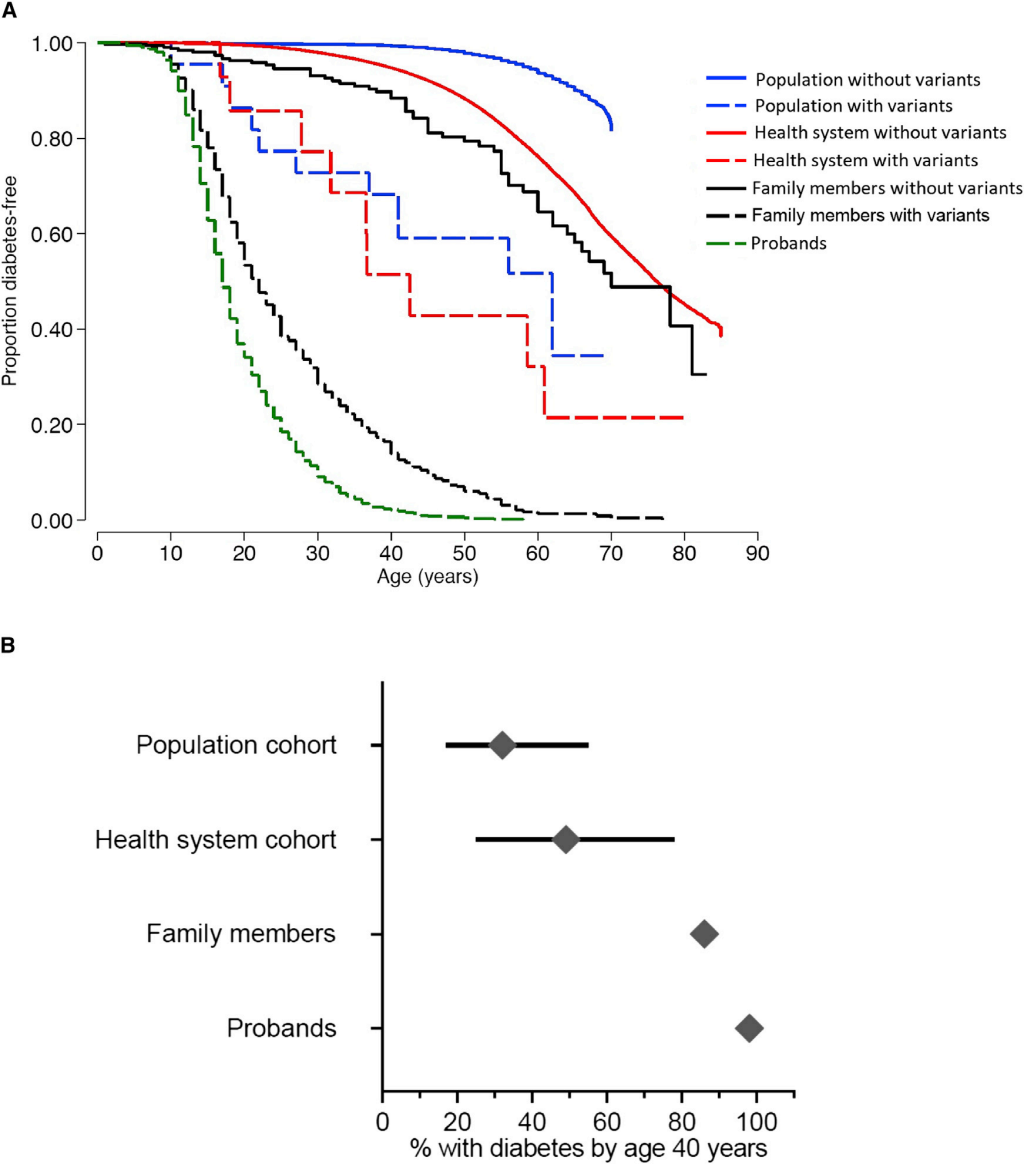
Penetrance of pathogenic *HNF1A* variants is lower in clinically unselected cohorts compared to a clinically ascertained cohort (A) Kaplan Meier survival curves of diabetes for individuals with (dashed line) and without *HNF1A* pathogenic variants (solid line) in four study cohorts. Analysis included probands (n = 661), their family members with (n = 622) and without (n = 332) *HNF1A* variants, individuals with (n = 14) and without (n = 132,180) *HNF1A* variants from Geisinger health system cohort, and individuals with (n = 22) and without (n = 198,726) *HNF1A* variants from UK Biobank population cohort. The log rank test p value for penetrance of diabetes for probands versus family member, Geisinger cohort, and UK Biobank cohort was 3 × 10^−26^, 3 × 10^−9^, and 5 × 10^−16^, respectively. (B) Penetrance of diabetes for individuals with pathogenic *HNF1A* variants in all four cohorts at age 40 years with 95% CI.

We assessed the age-related penetrance of pathogenic *HNF1A* variants in MODY probands (n = 661), their family members (n = 622), a health-system-based cohort (Geisinger cohort n = 132,194) and a population-based cohort (UK Biobank n = 198,748). The different background rate of diabetes highlights the different settings of these cohorts (100%, 61%, 24%, and 6%, respectively, Tables S3 and S4). Kaplan-Meier analysis demonstrated that the penetrance of diabetes for pathogenic *HNF1A* variants was lower in the family members, the Geisinger cohort, and UK Biobank compared to the *HNF1A*-MODY probands (log rank test, all p < 3 × 10^−9^; Figures 1A and S1). For example, by age 40 years, 98% (95%CI 97%–99%) of probands, 86% (83%–89%) of family members, 49% (25%–78%) of the Geisinger cohort, and 32% (17%–55%) of UK Biobank heterozygotes were diagnosed with diabetes, respectively (Figure 1B). The penetrance remained lower in these cohorts compared to probands after the adjustment of age, body mass index (BMI), sex, parental diabetes, and variant type (PTV vs. missense) in a multivariable Cox proportional hazard model (Table S5). The results were also similar when the analysis was restricted to unrelated individuals of European ancestry (Table S5). The analysis of pathogenic variants limiting to PTVs (assumed to have a similar biological impact because of nonsense-mediated decay) or limiting the MODY probands to missense pathogenic variants seen in unselected cohorts still showed lower penetrance in the unselected cohorts compared to probands (log-rank test all p < 1 × 10^−4^ and p < 2 × 10^−4^ respectively, Figures S2A and S3A–S3C).

### Penetrance of pathogenic *HNF4A* variants is lower in clinically unselected cohorts compared to a clinically ascertained cohort

**Figure 2:**
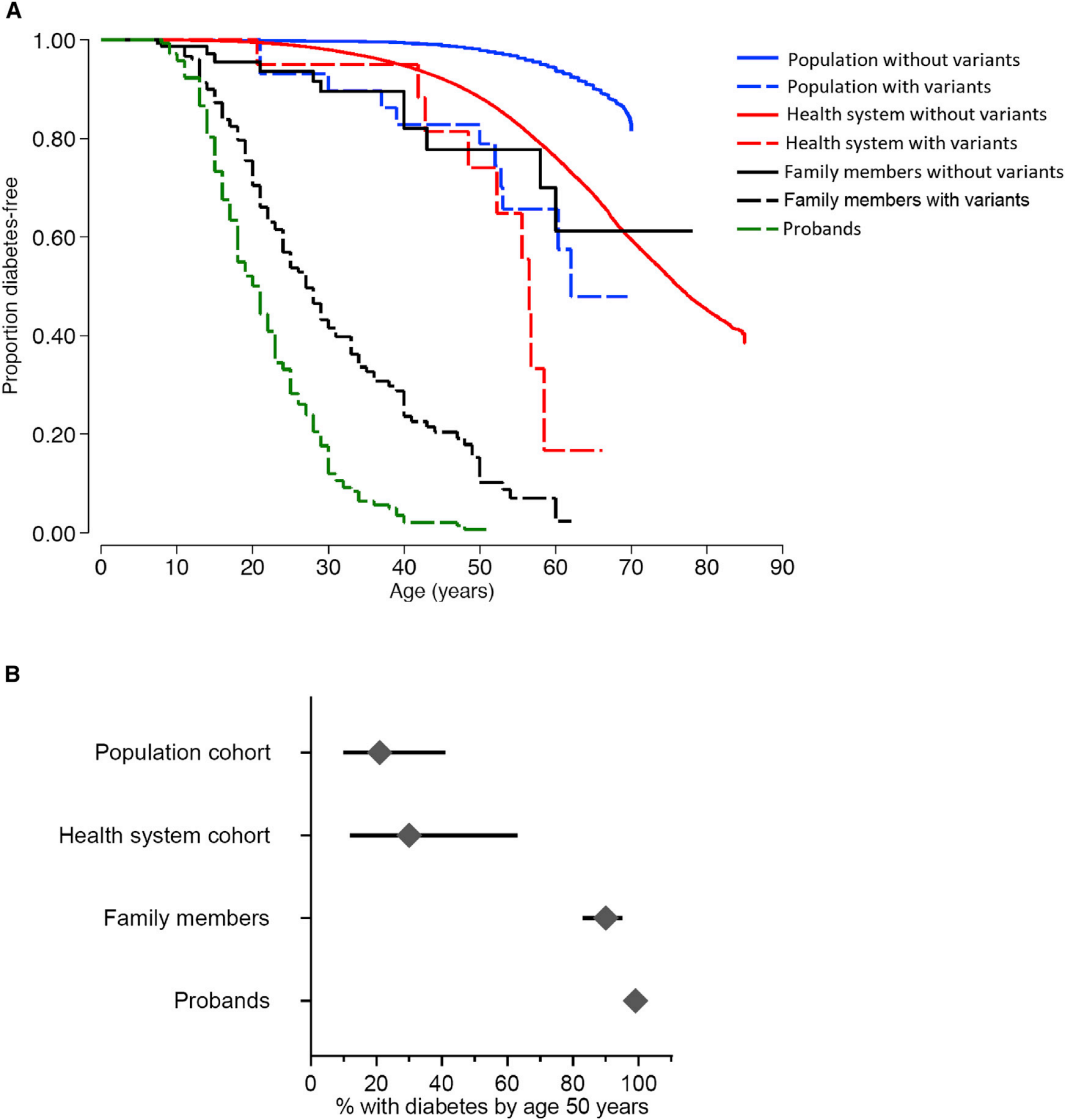
Penetrance of pathogenic *HNF4A* variants is lower in clinically unselected cohorts compared to a clinically ascertained cohort (A) Kaplan Meier survival curves of diabetes for individuals with (dashed line) and without (solid line) *HNF4A* variants in four study cohorts. Analysis included probands (n = 142), their family members with (n = 169) and without (n = 84) *HNF4A* variants, individuals with (n = 17) and without (n = 132,177) *HNF4A* variants from Geisinger health system cohort, and individuals with (n = 29) and without (n = 198,719) *HNF4A* variants from UK Biobank population cohort. The log rank test p value for penetrance of diabetes for probands versus family member, Geisinger cohort, and UK Biobank cohort was 8 × 10^−11^, 2 × 10^−12^, and 3 × 10^−19^, respectively. (B) Penetrance of diabetes for individuals with pathogenic *HNF4A* variants in all four cohorts at age 50 years with 95%CI.

Similar to *HNF1A*, for individuals with a *HNF4A* pathogenic variants (Tables S3 and S7), the age-related penetrance of diabetes was lower in the family members, the Geisinger cohort and the UK Biobank compared to MODY probands (log rank test, all p < 8 × 10^−11^; Figures 2A and S5). For example, by age 40 years, 98% (%CI 99%–100%) of probands, 76% (68%–85%) of family members, 5% (1%–31%) of the Geisinger cohort, and 17% (8%–37%) of UK Biobank heterozygotes developed diabetes. By age 50 years, 99% (95%CI 96%–100%) of probands, 90% (83%–95%) of family members, 30% (12%–63%) of the Geisinger cohort, and 21% (10%–41%) of UK Biobank heterozygotes developed diabetes (Figure 2B). The lower penetrance in clinically unselected cohorts was maintained after the adjustment of age, BMI, sex, parental diabetes, and variant type (PTV vs. missense) in a multivariable Cox proportional hazard model (Table S7). The result was also similar when the analysis was restricted to unrelated individuals of European ancestry (Table S7) or limiting the pathogenic variants to PTVs or limiting the MODY probands to missense pathogenic variants seen in unselected cohorts (log rank test all p < 0.01 and p < 0.0048 vs. probands, respectively, Figures S2B and S3D–S3F).

### Single-variant analysis suggested that reduced penetrance in clinically unselected cohort is not due to differences in pathogenic variants among these cohorts

In addition to the above sensitivity analysis of PTVs where biological impact is similar, we needed to assess the penetrance of a single pathogenic variant across the cohort to dispel the concern of differential effect of variants underlying the observed results. We did not have enough individuals of a single pathogenic variant in *HNF1A* or *HNF4A* for this analysis. However, we had adequate heterozygotes for the pathogenic but distinct *HNF4A* MODY subtype (including lower penetrance) caused by *HNF4A* c.340C>T (p.Arg114Trp) variant^30^ (n = 37, 43, 24, and 58 in MODY probands, proband family members, Geisinger, and UK Biobank exome-sequenced cohort, respectively). In line with our previous results, penetrance of diabetes in heterozygotes of *HNF4A* c.340C>T (p.Arg114Trp) variant was lower in the unselected cohorts compared to MODY probands (log rank test, all p < 1 × 10^−9^; Figure S5 and Table S8). These data together suggest that the lower penetrance of diabetes in the clinically unselected cohort is unlikely to be explained by the different pathogenic variants across these cohorts.

### The difference in the prevalence of diabetes in the study cohorts explains the difference in the age-dependent penetrance in heterozygotes of pathogenic *HNF1A/4A* variants

**Figure 3:**
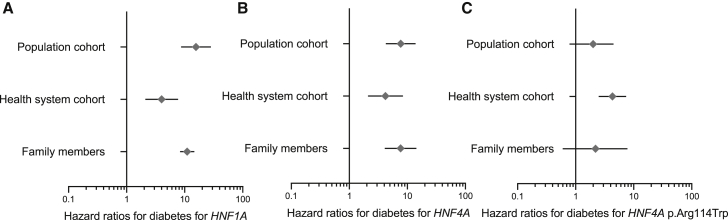
Cox proportional Hazard ratio for diabetes for individuals with pathogenic *HNF1A*, *HNF4A*, and *HNF4A* c.340C>T (p.Arg114Trp) variants relative to individuals without are largely similar in the clinically unselected cohorts (A) Graph showing the Cox proportional Hazard ratio and 95%CI for diabetes for individuals with relative to individuals without pathogenic *HNF1A* variants for family members, Geisinger health system cohort, and UK Biobank population cohort. (B) Graph showing the Cox proportional Hazard ratio and 95%CI for diabetes for individuals with relative to individuals without pathogenic *HNF4A* variants for same three cohorts. (C) Graph showing the Cox proportional Hazard ratio and 95%CI for diabetes for individuals with relative to individuals without pathogenic *HNF4A* c.340C>T (p.Arg114Trp) variant for same three cohorts.

The standard definition of penetrance (“the absolute risk of developing a disease in individuals with a pathogenic variant”)^11^ does not take into account the context in which individuals with the variant were identified. We hypothesized that the different settings in our study cohorts reflected by different rates of diabetes may explain the observed variation in the penetrance (absolute risk) of diabetes. We calculated Cox proportional hazard ratios (HR) for developing diabetes in *HNF1A/4A* heterozygotes relative to individuals without *HNF1A/4A* pathogenic variants in each of the three clinically unselected cohorts. HR for all three unselected cohorts were broadly similar for *HNF1A* heterozygotes albeit slightly lower in the Geisinger cohort (11 [95%CI 8–15] for family members, 4 [2–8] for Geisinger cohort, 16 [9–28] for UK Biobank) (Figure 3A and Table S9). Similar results were seen for *HNF4A* heterozygotes (8 [4–14] for family members, 4 [2–8] for Geisinger cohort, 8 [4–14] for UK Biobank, Figure 3B) and individuals with *HNF4A* c.340C>T (p.Arg114Trp) variant (1 [0.6–3], 3 [2–5], 2 [0.8–4] respectively, Figure 3C and Table S9). We observed similar results when the analyses were limited to unrelated individuals of European ancestry (Table S9). These data together suggest that pathogenic variants have a similar effect within each unselected cohort; the different absolute risks of diabetes in these cohorts can largely be explained by a different proportion of non-variant related factors (genetic or nongenetic modifiers) that may contribute to the risk of diabetes (i.e., highest in family members and lowest in UK Biobank).

### Penetrance of pathogenic *GCK* variants is not affected by different settings of cohort

We next assessed whether the penetrance of pathogenic *GCK* variants was influenced by the background rates of diabetes similar to those of *HNF1A*/*HNF4A*-MODY. In contrast to the age-related phenotype of *HNF1A*/*HNF4A*-MODY, *GCK*-MODY causes life-long stable mild fasting hyperglycemia from birth with a modest increase with age rather than true progressive diabetes as seen in *HNF1A/HNF4A*-MODY.^7^^,^^31^^,^^32^ Therefore, its penetrance is assessed by the presence of mild hyperglycemia (defined in this study as fasting blood glucose ≥ 5.6 mmol/L and/or HbA1c ≥ 39 mmol/mol)^20^ (Tables S3 and S10).

**Figure 4:**
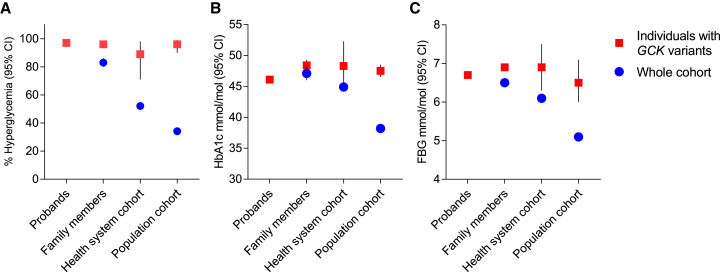
Penetrance of mild hyperglycemia for pathogenic *GCK* variants is similar in clinically selected and unselected cohorts (A) Proportion and 95%CI of individuals with mild hyperglycemia (HbA1c ≥ 39 mmol/mol and/or a fasting glucose ≥5.6 mmol/L, definition of prediabetes by American Diabetes Association) for probands of pathogenic *GCK* variants (n = 939) and individuals with pathogenic *GCK* variants in their family members (n = 723), Geisinger heath system cohort (n = 32), and UK biobank population cohort (n = 83) as red square. Background rate of mild hyperglycemia and 95%CI in each unselected cohort is also shown as blue circle. (B) Mean HbA1c and 95%CI for *GCK*-MODY probands and individuals with pathogenic *GCK* variants in their family members, Geisinger cohort, and UK Biobank as red square. Cohort mean HbA1c and 95%CI for each unselected cohort is shown as blue circle. (C) Mean fasting blood glucose and 95%CI for *GCK*-MODY probands and individuals with pathogenic *GCK* variants in their family members, Geisinger cohort, and UK Biobank as red square. Cohort mean fasting blood glucose and 95%CI for each unselected cohort is shown as blue circle.

The penetrance of mild hyperglycemia was 97% (95%CI 96%–98%) for *GCK*-MODY probands. Unexpectedly, the penetrance of *GCK* heterozygotes in the unselected cohorts was similar to probands (family members 96% [94%–98%], p = 0.30, Geisinger cohort 89% [71%–98%], p = 0.04, UK Biobank 96% [90%–99%], p = 0.48) despite the difference in the prevalence of mild hyperglycemia (83%, 52%, and 34%, respectively) in the clinically unselected cohorts (Figure 4A). Similarly, the mean HbA1c of *GCK-MODY* probands was similar to those with *GCK* pathogenic variants from the Geisinger cohort (46.1 [95%CI 45.7–46.6] vs. 48.3 [44.3–52.3], p = 0.1) and UK Biobank (47.5 [46.5–48.5], p = 0.09) and marginally lower than that of family members (48.4 [47.5–49.3], p < 0.0001 unadjusted, and p > 0.05 for after adjustment of age, sex, and BMI) (Figure 4B and Table S11). This is despite the lower and different background levels of HbA1c across the clinically unselected cohorts (mean HbA1c of 47.1, 44.9, and 38.2 mmol/L, respectively, p < 0.0001). Fasting blood glucose analysis also showed similar results as HbA1c (Figure 4C and Table S12). Analyses restricting to unrelated individuals of European ancestry also showed equivalent results for HbA1c and fasting blood glucose (Tables S11, S12, and S13).

## Discussion

Using four cohorts with different settings and >300,000 individuals, we show that pathogenic variants in the three MODY-associated genes are not rare in the population. Pathogenic variants in *HNF1A* and *HNF4A* show the highest risk of diabetes in clinically selected individuals but had substantially lower risk in clinically unselected settings. We show that this lower risk was not attributed to the types of variants but rather to the characteristics of the setting in which they were identified. Surprisingly, the penetrance of pathogenic *GCK* variants was similar irrespective of the setting of the cohorts.

Our study substantially contributes to the existing evidence of the prevalence and penetrance of diabetes of the three most common MODY-associated genes. Our study provides an accurate and comprehensive estimate of the risk of diabetes in different settings and its prevalence in the population. We estimate that up to 1 in 1,500 individuals contain a pathogenic variant in the three most common MODY-associated genes (covers >80% of MODY) in the population. This is higher than the estimate from the previous studies which reported the prevalence of MODY from 1:4,032 to 9,239 in the UK population.^3^^,^^33^ However, the estimates from these studies were based on individuals with clinically suspected MODY (early-onset diabetes) who were referred for genetic testing from routine clinical practice. They essentially estimate the prevalence of a certain phenotype with a pathogenic variant whereas our estimate is irrespective of diabetes status and clinical selection. Our estimate of the prevalence of pathogenic *GCK* variants ranges from 1:4,100 to 1:2,400. This was slightly lower than the previous estimate of 1:1,000; however, that study selected individuals based on fasting glucose and had a small sample size of 5,500 and had a wide confidence interval of the estimates (1:344 to 1:3,333).^34^

We provide comprehensive estimates of diabetes risk in clinically selected individuals as well as in three different clinically unselected scenarios. We show that diabetes risk is substantially lower in clinically unselected settings for *HNF1A*/*HNF4A*-MODY but not for *GCK*-MODY. The different settings of our unselected cohort ("unhealthy volunteer" selection bias [Geisinger cohort]^35^ to "healthy volunteer" selection bias [UK Biobank]^36^) are likely to cover the most common situations where incidental pathogenic variants will be identified. Outside of our clinically referred to cohort, we found that most individuals with *HNF1A*- and *HNF4A*-MODY did not develop diabetes even by 50 years of age (∼50% and ∼80% in Geisinger and UK Biobank, respectively). The large sample size of our study, robust unbiased phenotyping, replication in an unselected cohort with Sanger confirmation, similar results with protein-truncating variants, and a single *HNF4A* variant suggest that lower penetrance in the unselected setting is less likely due to variant heterogeneity or false-positive variants. The lack of enrichment of prediabetes in individuals with *HNF1A* and *HNF4A* variants in unselected cohort suggest that these individuals do not have enrichment of milder subclinical disease (Table S14). The previous studies did observe similar results, but they were limited by sample size (unselected n < 39,000), had a small number of people with pathogenic variants (≤5 in *HNF1A*), and lacked a clinically selected cohort to robustly support the conclusion.^12^^,^^13^ A recent study with a single pathogenic variant in *HNF1A* also found lower penetrance in extended family members supporting our results in the population cohort.^37^ Our results showing reduced risk of disease in clinically unselected individuals have important clinical implications for the incidental finding of monogenic diabetes and a wide range of other monogenic disorders. Our data suggest that intensive close monitoring of the onset of diabetes may not be necessary for people with incidentally identified pathogenic variants in *HNF1A* and *HNF4A* due to a substantially lower risk of diabetes. Our study also highlights the caution necessary when using disease-variant association in a population cohort to downgrade the pathogenicity of a variant of interest as the lack of association may simply be a reflection of the lower penetrance in the population cohort.

The reduced penetrance of *HNF1A*/*HNF4A*-MODY in clinically unselected cohorts was largely consistent with the setting in which they were identified. The penetrance (absolute risk) of diabetes was highest in the family members of probands followed by the health system-based cohort (Geisinger) and lowest in the population-based cohort (UK Biobank). Interestingly, the relative risk (hazard ratios) of getting diabetes was largely constant across three cohorts. These suggest that the inherent risk of diabetes with pathogenic variants is constant irrespective of the setting and that the variation in the observed absolute risk of diabetes is due to varying proportions of non-variant factors (environmental or/and genetic modifiers).^11^^,^^37^ These results also suggest that these factors are proportionally more in family members and less in the UK Biobank participants. Further studies across different settings are needed to identify genetic or non-genetic modifiers. A recent study of inherited cardiomyopathy observed a similar finding of variable absolute risks in different settings but comparable relative risks.^38^ Our study also suggests that relative risk may be a better reflection of the biological effect (pathogenicity) of the rare variants and represent a better parameter to classify high- and low-penetrance variants/gene compared to the absolute risk. However, this may not be true for all monogenic diseases.

The penetrance of the *GCK* pathogenic variants is very high irrespective of the setting. This was previously observed in a smaller cohort (N = 77,184)^12^ and may reflect the pathogenic mechanisms of *GCK*-MODY. *GCK* acts as a glucose sensor for the beta cells. It has unique catalytic properties including a markedly lower affinity for glucose and a lack of significant feedback inhibition.^39^^,^^40^ The combination of these means the rate of glucose phosphorylation is proportional to the glucose concentration in the blood leading to maintenance of the glucose at very tight glucose values (4–5 mmol/L).^41^ The mutations in *GCK* reduce the function of glucokinase which leads to an upward shift at which the glucose is regulated.^31^ This simply means the body’s physiological metabolic response remains normal but it resets to maintain the glucose at a higher level (i.e., same level of insulin but at higher glucose compared to controls) leading to physiologically regulated life-long stable mildly higher glucose values of 5.5–8 mmol/L with only a modest increase with age.^7^^,^^31^ Because of this, pharmacological treatment has no impact on the glucose level.^6^ This, along with a lack of diabetes-related complications, means these individuals do not need treatment for their mild hyperglycemia.^31^ Despite these clear clinical implications, these individuals are often mistreated as type 1 or type 2 diabetes.^42^^,^^43^ The high penetrance irrespective of setting, the high frequency in the population (∼1:2,000), and the unnecessary treatment and follow-up make *GCK* a good candidate gene for ACMG secondary list to avoid unnecessary treatment for diabetes in these individuals.

We were limited by the number of individuals with pathogenic variants in *HNF1A* and *HNF4A* in unselected cohorts. This is mainly a reflection of the rarity of the monogenic disease in the population and our strict criteria of defining pathogenicity in these cohorts. The replication of our results in two independent cohorts and unbiased assessment of diabetes status irrespective of the pathogenic variant status is a real strength of our study, which we believe strongly overcomes the limited number of participants in our study. Our study is limited by the lack of Sanger sequencing confirmation of variants in the UK Biobank. However, the use of our robust quality filters and manual check of the IGV (Integrative Genomics Viewer) plots for all the pathogenic variants means the false positive variants are extremely unlikely to have been included in the analyses. Our study was also limited by having an unselected cohort significantly older than the probands. We used Kaplan-Meier analysis and Cox proportional hazard models to address this in our analysis. However, it will still not completely address this issue. We believe that the older age of unselected cohort will lead to higher penetrance, not the lower penetrance that we observed in the unselected cohort, because there is more time to develop diabetes. Equally, it is also possible that some of the people with early-onset diabetes are not present in the unselected cohort particularly in the UK Biobank, which has “healthy volunteer” bias.^36^ This may have lowered the observed penetrance.

Our results demonstrate that pathogenic variants in common MODY-associated genes are not ultra rare in the population, and they show substantially reduced penetrance of diabetes when identified incidentally. We highlight the need to tailor genetic interpretation and counseling based on the setting, the family history, and health status when reporting actionable genetic variants identified incidentally, because penetrance is substantially reduced in unselected settings. However, *GCK*-MODY is an exception which, despite being relatively not rare in the population, shows near-complete penetrance irrespective of the setting, making it an excellent candidate for the ACMG secondary gene list to avoid unnecessary treatment of individuals with this monogenic diabetes.

## Data Availability

The data supporting the findings of this study are available within the article and its supplemental information. Additional information for reproducing the results described in the article is available upon reasonable request and subject to a data use agreement. The UK Biobank dataset is available from https://biobank.ctsu.ox.ac.uk. All variant assertion scores and classification in the unselected cohorts have been submitted to ClinVar (accession numbers SCV002562106–SCV002562202).

## Supplemental information

Document S1. Figures S1–S5, Tables S1, S2, and S4–S14, and consortium details

Table S3. Pathogenic variants of HNF1A, HNF4A, and GCK in MODY proband, proband family members, Geisinger cohort, and UK Biobank

Document S2. Article plus supplemental information
