# Supplementary material for: Reduced penetrance of MODY-associated HNF1A/HNF4A variants but not GCK variants in clinically unselected cohorts
Source: Am J Hum Genet. 2022 Oct 17;109(11):2018–28. doi: 10.1016/j.ajhg.2022.09.014 (PMC9674944; doi:10.1016/j.ajhg.2022.09.014)
Supplement: Document S1. Figures S1–S5, Tables S1, S2, and S4–S14, and consortium details [file mmc1.pdf]

The American Journal of Human Genetics, Volume *109*

Supplemental information

Reduced penetrance of MODY-associated *HNF1A*/*HNF4A* variants but not *GCK* variants in clinically unselected cohorts

Uyenlinh L Mirshahi, Kevin Colclough, Caroline F Wright, Andrew R Wood, Robin N Beaumont, Jessica Tyrrell, Thomas W Laver, Richard Stahl, Alicia Golden, Jessica M Goehringer, Geisinger-Regeneron DiscovEHR Collaboration, Timothy F Frayling, Andrew T Hattersley, David J Carey, Michael N Weedon, and Kashyap A Patel

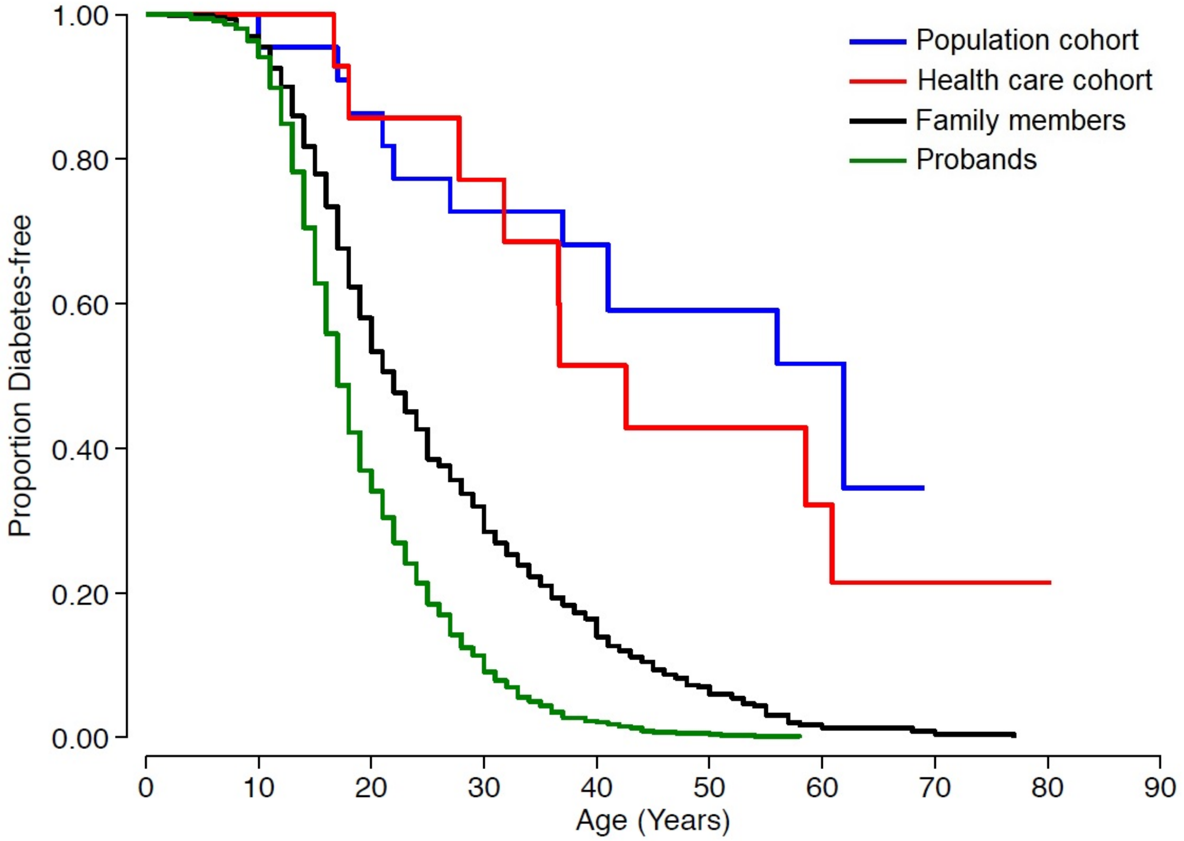

Figure S1: Penetrance of diabetes for individuals with pathogenic *HNF1A* variants in clinically selected and unselected cohorts. Kaplan-Meier survival curves of diabetes for *HNF1A*-MODY probands (N=661), their family members with pathogenic *HNF1A* variants (N=622), and individuals with pathogenic *HNF1A* variants from health care-based Geisinger cohort (N=14) and UK Biobank population cohort (N=22). The log rank test p values for probands versus each unselected cohort were $3x10^{-26}$, $3x10^{-09}$, $5x10^{-16}$, respectively.

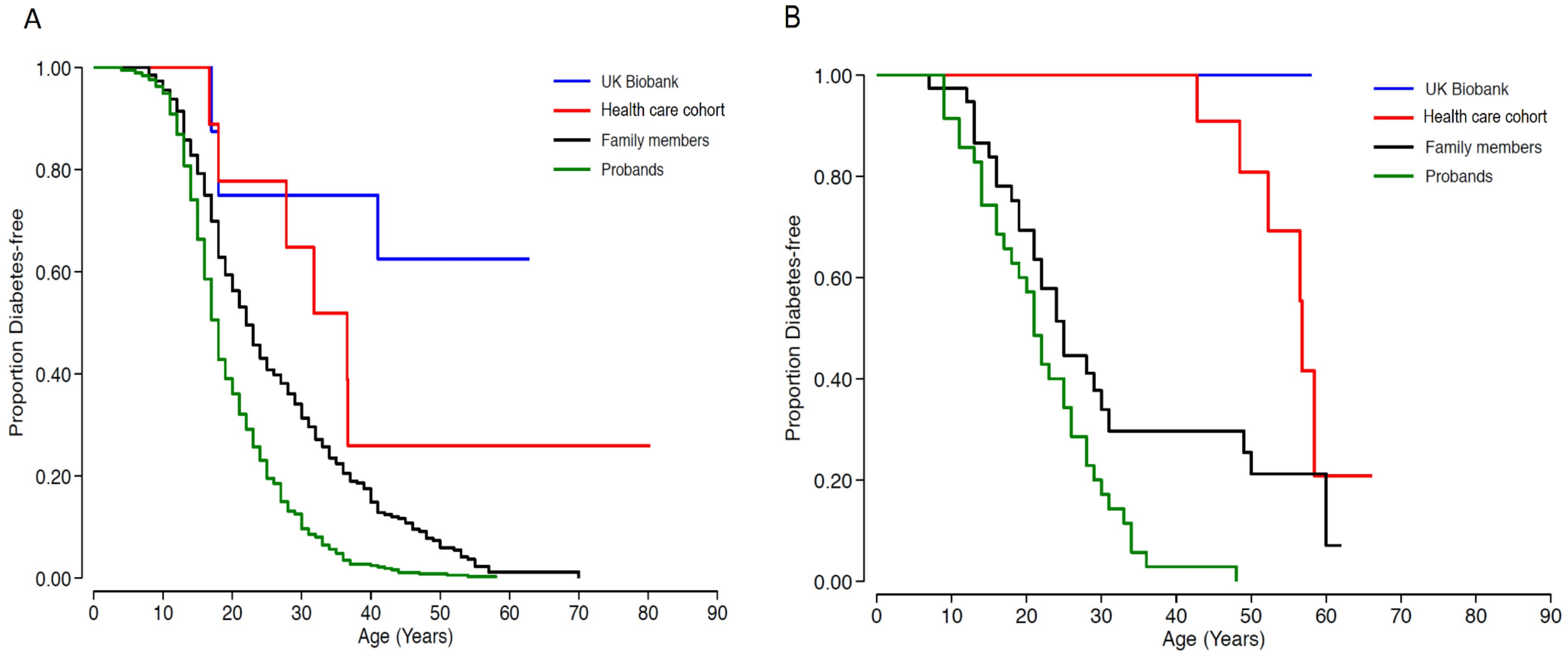

Figure S2. Penetrance of diabetes for *HNF1A* and *HNF4A* protein truncating variants (PTVs) observed in individuals from clinically selected and unselected cohorts. Kaplan-Meier survival curves of diabetes for A) *HNF1A*-MODY in probands (N=374), their family members (N=350), healthcare-based Geisinger cohort (N=9), and UK Biobank population cohort (N=8) restricted to PTVs seen in all those cohorts only. The log rank test p values verses probands for each unselected cohort were $2\text{x}10^{-14}$, $1\text{x}10^{-04}$, $2\text{x}10^{-07}$, respectively. B) Same as A but for *HNF4A*-MODY; probands (N=35), their family members with *HNF4A* PTVs (N=43), and individuals with *HNF4A* PTVs from healthcare-based Geisinger cohort (N=12) and UK Biobank population cohort (N=4). The log rank test p values verses probands for each unselected cohort were 0.01, $2\text{x}10^{-08}$, 0.0002, respectively.

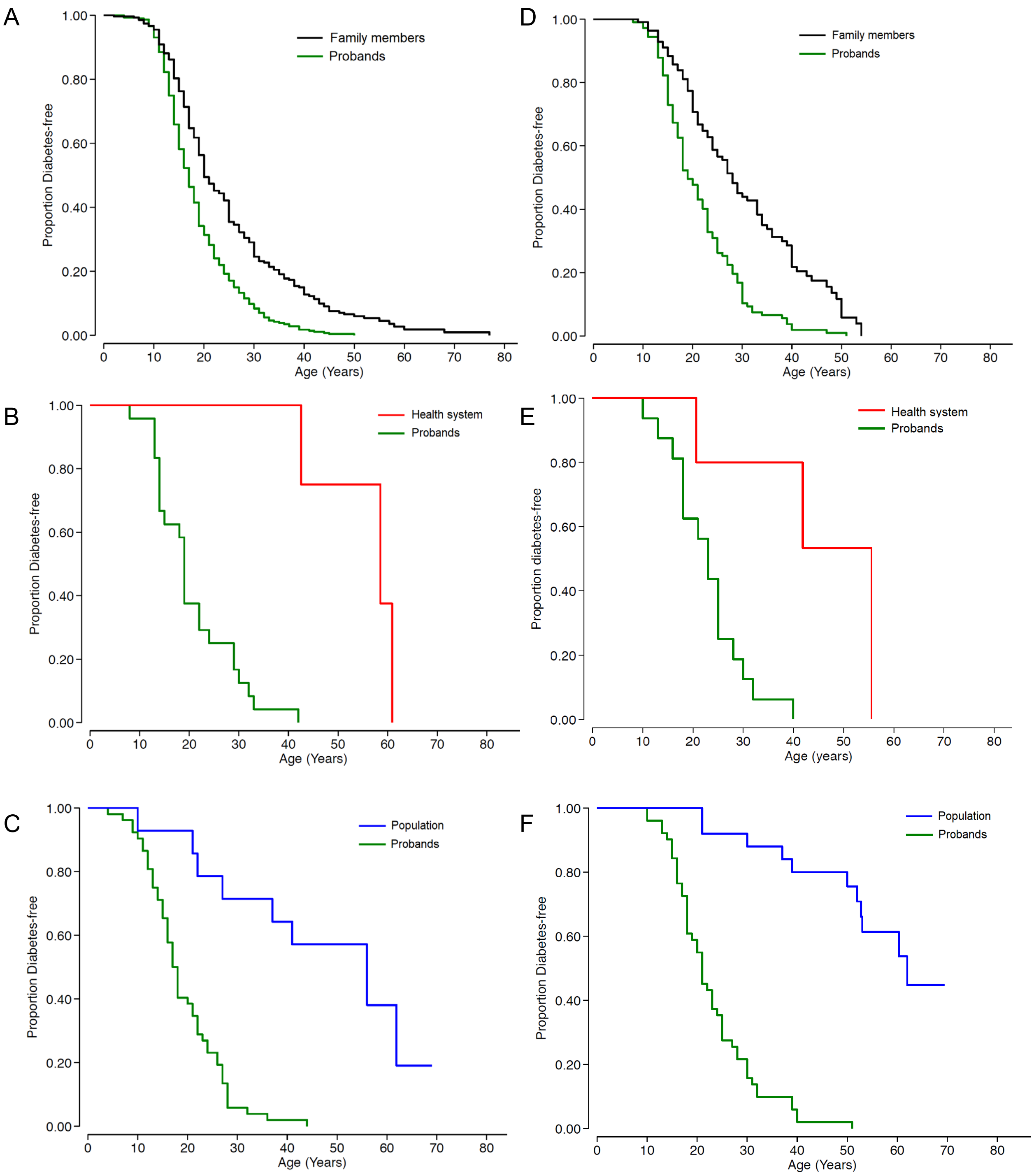

Figure S3. Penetrance of diabetes restricted to individuals with missense variants in *HNF1A* and *HNF4A* from unselected comparison cohort and probands**.** Kaplan-Meier survival curves of diabetes for *HNF1A*-MODY for A) proband family members (N=272) vs. probands (N=287), B) Geisinger healthcare system (N=5) vs. probands (N=287), and C) UK Biobank population (N=14) vs. probands (N=287). The log rank test p values verses probands for each unselected cohort were 3.2x10^{-12}, 2.3x10^{-04}, 5.9x10^{-07}, respectively. Kaplan Meier survival curves of diabetes for *HNF4A*-MODY for D) proband family members (N=126) vs. probands (N=107), E) Geisinger (N=5) vs. probands (N=16), and F) UK Biobank (N=25) vs. probands (N=51). The log rank test p values verses probands for each unselected cohort were 3.9x10^{-09}, 4.8x10^{-03}, 7.4x10^{-13}, respectively.

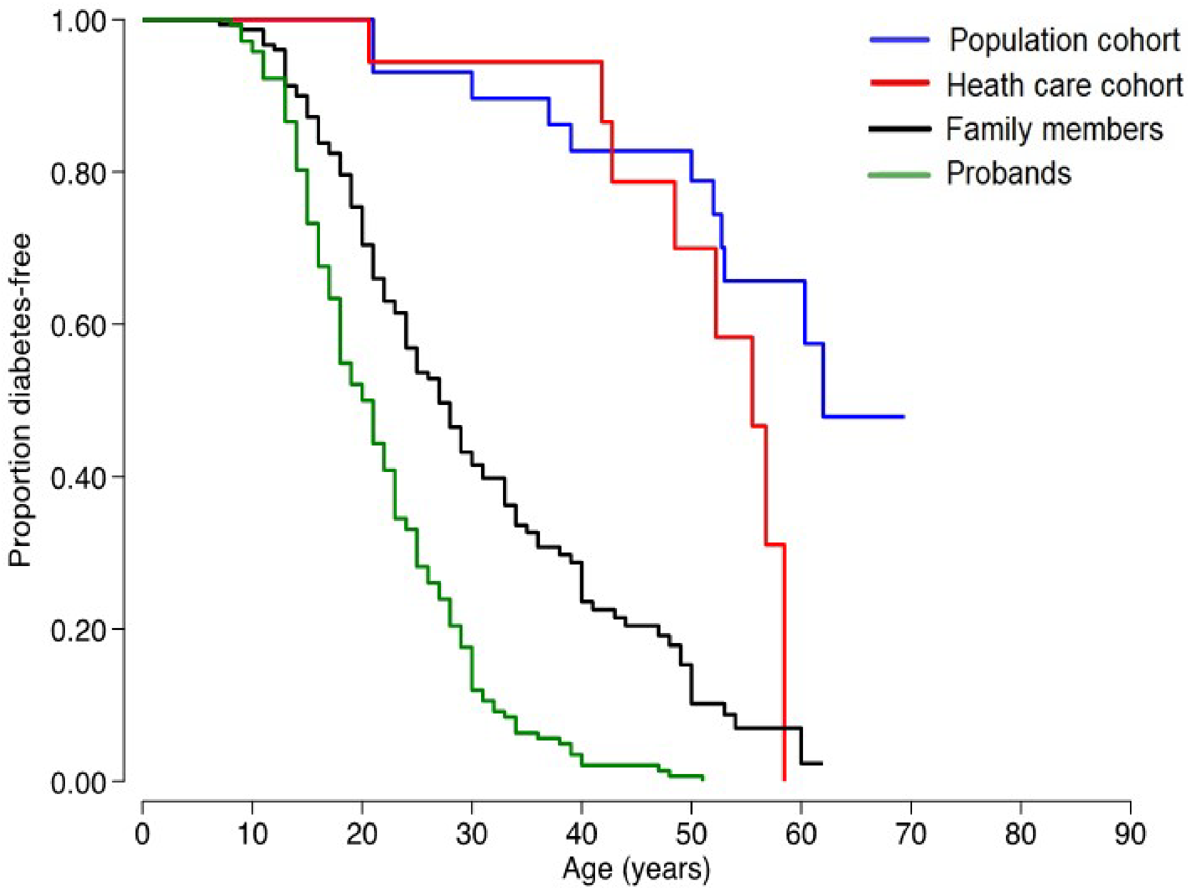

Figure S4. Penetrance of diabetes for individuals with pathogenic *HNF4A* variants in clinically selected and unselected cohorts. Kaplan-Meier survival curves for diabetes for *HNF4A*-MODY probands (N=142, their family members with pathogenic *HNF4A* variants (N=169), and individuals with pathogenic *HNF4A* variants in health care-based Geisinger cohort (N=17) and UK Biobank population cohort (N=29). The log rank test p values probands versus each unselected cohort were $8x10^{-11}$, $2x10^{-12}$, $3x10^{-19}$, respectively.

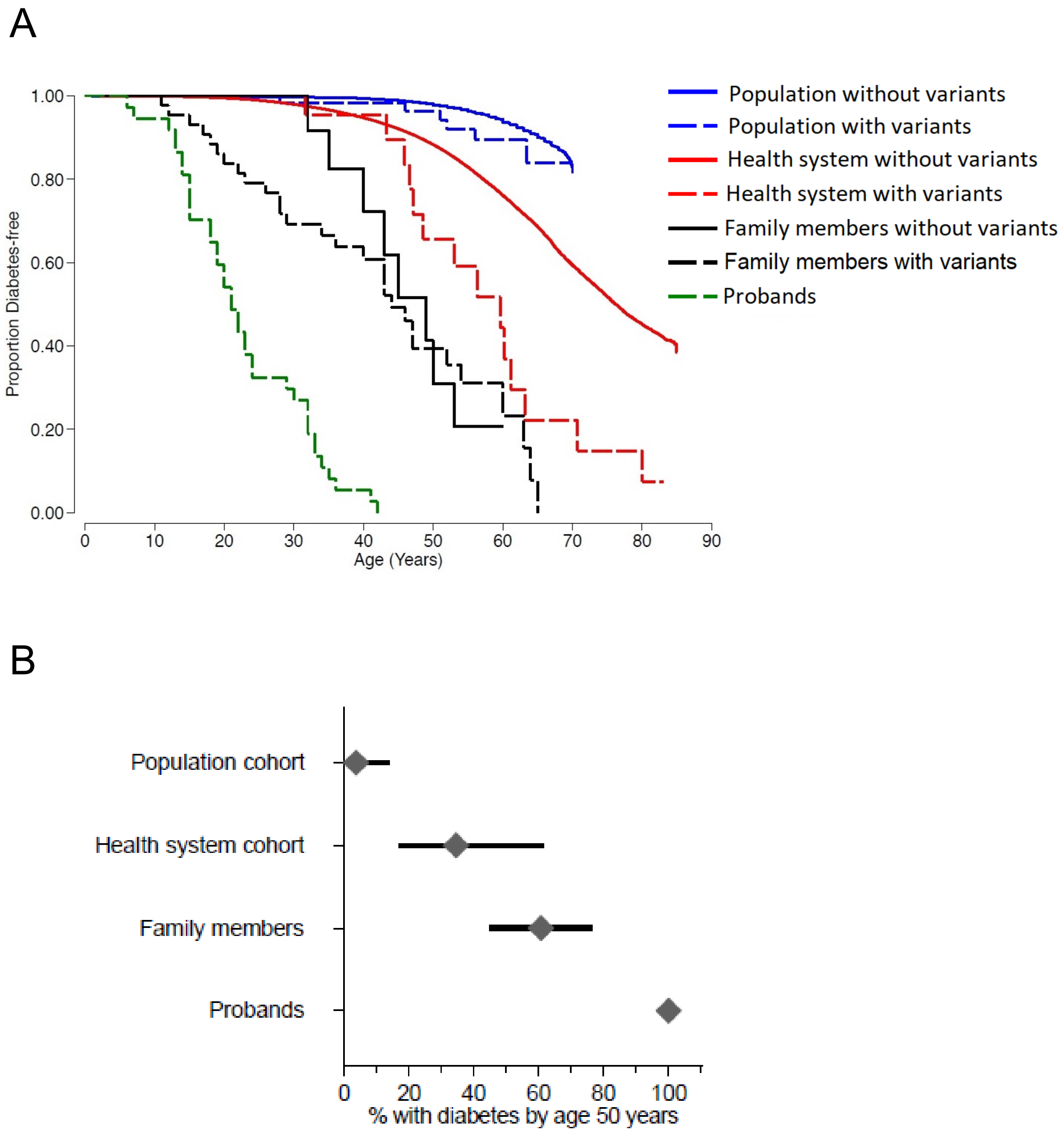

Figure S5. Penetrance of diabetes for individuals with and without pathogenic *HNF4A* p.Arg114Trp variant in clinically selected and unselected cohorts. A) Kaplan Meier survival curves of diabetes for *HNF4A*-MODY p.Arg114Trp pathogenic variant probands (N=37), their family members with (N=43) and without (N=41) *HNF4A* p.Arg114Trp, individuals with (N=24) and without (N=132,170) *HNF4A* p.Arg114Trp from Geisinger healthcare system cohort, and individuals with (N=58) and without (N=198,690) *HNF4A* p.Arg114Trp from UK Biobank population cohort. The log rank test p value for probands versus family members was $1x10^{-09}$, individuals from Geisinger cohort was $1x10^{-13}$, and individuals from UK Biobank $5x10^{-29}$. Results were similar when analysed with unrelated individuals of European ancestry with and without adjustment for age at study, sex, BMI, parents with diabetes status, and variant types (see Table S8). B) Penetrance of diabetes for individuals with pathogenic *HNF4A* p.Arg114Trp variant in all four cohorts at age 50 years with 95% CI

Characteristics	MODY probands (index cases)	MODY family members	Geisinger cohort	UK Biobank
**N**	1,742	2,194	132,194	198,748
**Age, y**	27.3 (14.6)	35.1 (20.3)	52.9 (17.5)	56.9 (8.1)
**Female Sex, n (%)**	1,141 (66)	1,291 (59)	80,956 (61)	109,387 (55)
**BMI at recruitment (kg/m^2)**	23.9 (4.1), n=1,360	25.2 (4.4), n=1,016	31.4 (8.2), n=129,529	27.4 (4.7), n=197,798
**Diabetes*, n (%)**	803 (100)	1,168 (53)	31,266 (24)	11,488 (6)
**Age at diabetes diagnosis, y**	19.5 (9.4)	28.0 (15.3)	52.6(14.4)	52.5 (12.0)
**Parent with diabetes, n (%)**	1,393 (80)	1,306 (60)	40,393 (31)	34,270 (17)
**HbA1c, mmol/mol**	52.5 (15.7), n=1,439	49.8 (17.1), n=984	44.9 (15.3), n=58,920	38.2 (6.2), n=188,924
**Fasting glucose, mmol/l**	7.1 (1.9), n=958	6.5 (2.1), n=655	6.1 (2.2), n=85,437	5.1 (1.0), n=41,898
**European ancestry, n (%)**	1,437 (90)	1,776 (94)	125,850 (95)	182,920 (92)
**Unrelated, n (%)**	1,742 (100)	814 (37)	87,234 (66)	184,142 (93)

Table S1: Characteristics of the study cohorts at recruitment. Values are mean (SD) for continuous variables or number of individuals (%) for categorical variables. The number of individuals with available data is also indicated where appropriate. Abbreviations MODY, matured onset diabetes of the young; y, years; BMI, body mass index, *excluding *GCK*-MODY probands

Characteristics	*HNF1A*	*HNF4A*	*GCK*
**N**	954	253	987
**Age at recruitment, y**	36.6 (19.1)	24.3 (20.9)	33.9 (21.3)
**Female Sex, n (%)**	562 (59)	154 (61)	575 (58.3)
**BMI at recruitment (kg/m^2)**	25.2 (4.2), n=536	24.3 (20.9), n=253	25.1 (4.7), n=370
**Diabetes, n (%)**	586 (61)	125 (49)	457 (46.3)
**Age at diabetes diagnosis, y**	25.7 (13.8)	27.1 (12.6)	32.1 (17.4)
**Parent with diabetes, n (%)**	681 (71)	182 (72)	443 (44.9)
**HbA1c, mmol/mol**	50.9 (20.4), n=486	55.6 (17.4), n=89	47.1 (11.1), n=409
**Fasting glucose, mmol/l**	6.7 (3.1), n=199	5.9 (2.6), n=34	6.5 (1.3), n=422
**European Ancestry, n (%)**	840 (95)	190 (90)	746 (93.4)
**Unrelated, n (%)**	328 (34)	86 (34)	400 (40.5)

Table S2: Characteristics of all family members at recruitment by each gene. Values are mean (SD) for continuous variables or number of individuals (%) for categorical variables. The number of individuals with available data is also indicated where appropriate.

Table S3: Pathogenic variants of *HNF1A*, *HNF4A*, and *GCK* in MODY proband, proband family members, Geisinger cohort, and UK Biobank. This table is included in Excel format.

Characteristics	MODY probands (index cases)	MODY family members	Geisinger cohort	UK Biobank
**N**	661	622	14	22
**Age, y**	31.7 (15.0)	36.8 (18.3)	47.8 (20.1)	56.1 (8.5)
**Female Sex, n (%)**	455 (69)	366 (59)	10 (71)	14 (64)
**BMI (kg/m^2)**	24.6 (4.0), n=527	24.8 (3.9), n=367	27.3 (6.9), n=14	26.2 (4.1), n=22
**Diabetes, n (%)**	661 (100)	526 (85)	9 (64)	11 (50)
**Diagnosed with diabetes by biomarkers only, n (%)**	---	---	0	1 (4.5)
**Age at diabetes diagnosis, y**	19.3 (7.8)	23.9 (11.8)	36.6 (15.6)	32.0 (16.8)
**Parent with diabetes, n (%)**	582 (88)	491 (79)	7 (58)	14 (64)
**HbA1c, mmol/mol**	59.2 (19.0), n=553	56.5 (19.1), n=355	56.3 (12.4), n=10	46.4 (12.7), n=21
**Fasting glucose, mmol/l**	8.3 (3.1), n=193	7.7 (3.6), n=119	7.7 (2.7), n=8	5.1 (1.1), n=5
**European ancestry, n (%)**	564 (90)	546 (93)	14 (100)	22 (100)
**Unrelated, n (%)**	661 (100)	250 (40)	12 (86)	21 (95)
**PTV, n (%)**	374 (57)	350 (56)	9 (64)	8 (36)
**Missense, n (%)**	287 (43)	272 (44)	5(36)	14 (64)

Table S4: Characteristics of *HNF1A* heterozygotes in MODY probands, proband family members, Geisinger cohort, and UK Biobank at recruitment. Values are mean (SD) for continuous variables or number of individuals (%) for categorical variables. The number of individuals with available data is also indicated where appropriate. Abbreviations MODY, matured onset diabetes of the young; y, years; BMI, body mass index; PTV, putative truncating variants

	Unadjusted			Adjusted*		
	**Hazard Ratio (95% CI)**	**P Value**	**N**	**Hazard Ratio (95% CI)**	**P Value**	**N**
**All individuals**						
**Probands**	Base		661	Base		527
**Family members of proband**	0.6 (0.5 - 0.6)	1.1E-23	622	0.7 (0.6 - 0.9)	5.7E-05	367
**Geisinger cohort**	0.2 (0.1 - 0.3)	7.7E-07	13	0.2 (0.1 - 0.4)	6.2E-05	13
**UK Biobank**	0.1 (0.1 - 0.2)	2.8E-11	22	0.2 (0.1 - 0.3)	5.5E-07	22
**Unrelated Europeans**						
**Probands**	Base		564	Base		456
**Unrelated Family members of proband**	0.5 (0.4 - 0.6)	3.8E-17	219	0.8 (0.6 - 1.0)	0.04	134
**Unrelated Geisinger cohort**	0.1 (0.1 - 0.3)	1.7E-06	12	0.2 (0.1 - 0.4)	9.8E-05	12
**Unrelated UK Biobank**	0.1 (0.1 - 0.2)	2.0E-10	21	0.2 (0.1 - 0.4)	1.8E-06	21

Table S5: Univariate and multivariate Cox proportional hazard ratios for diabetes for individuals with pathogenic *HNF1A* variants in each unselected cohort relative to *HNF1A*-MODY probands. Hazard ratios (95% CI), p values, and number of all individuals with pathogenic variants (top) and unrelated individuals of European ancestry with pathogenic variants (bottom) in each cohort in univariate (left) and multivariate (right) regression analysis vs. probands (base). *Multivariate regression adjusted for age at recruitment, sex, body mass index, family history of diabetes, and variant type (PTV vs. missense). Abbreviations CI, confidence intervals

Characteristics	MODY probands (index cases)	MODY family members	Geisinger cohort	UK Biobank
**N**	142	169	17	29
**Age, y**	33.1 (14.6)	35.8 (21.0)	46.8 (13.6)	56.8 (7.1)
**Female Sex, n (%)**	103 (73)	101 (60)	7 (41)	18 (62)
**BMI (kg/m2)**	25.5 (4.6), n=127	25.7 (4.9), n=89	31.6 (5.1), n=17	26.8 (4.4), n=28
**Diabetes, n (%)**	142 (100)	114 (67)	8 (47)	11 (38)
**Diagnosed with diabetes by baseline biomarkers only, n (%)**	---	---	1 (5.6)	1 (3.4)
**Age at diabetes diagnosis, y**	21.6 (8.3)	26.6 (12.0)	47.1 (12.4)	43.5 (14.7)
**Parent with diabetes, n (%)**	122 (86)	139 (82)	5 (29)	13 (45)
**HbA1c, mmol/mol**	62.4 (22.2), n=118	57.4 (16.9), n=74	50.0 (14.5), n=12	45.3 (12.4), n=27
**Fasting glucose, mmol/l**	9.5 (3.8), n=38	6.4 (3.0), n=23	7.8 (4.6), n=15	4.8 (0.7), n=7
**European ancestry, n (%)**	122 (89)	131 (90)	17 (100)	26 (90)
**Unrelated, n (%)**	142 (100)	66 (39)	15 (88)	27 (93)
**PTV, n (%)**	35 (25)	43 (25)	12 (71)	4 (14)
**Missense, n (%)**	107 (75)	126 (75)	5 (29)	25 (86)

Table S6: Characteristics of *HNF4A* heterozygotes in MODY probands, proband family members, Geisinger cohort and UK Biobank at recruitment. Values are mean (SD) for continuous variables or number of individuals (%) for categorical variables. The number of individuals with available data is also indicated where appropriate. Abbreviations MODY, matured onset diabetes of the young; y, years; BMI, body mass index; PTV, putative truncating variants.

	Unadjusted			Adjusted*		
	**Hazard Ratio (95% CI)**	**P Value**	**N**	**Hazard Ratio (95% CI)**	**P Value**	**N**
**All individuals**						
**Probands**	Base		142	Base		127
**Family members of proband**	0.4 (0.3 - 0.6)	$8x10^{-10}$	169	0.7 (0.5 - 1.0)	0.03	89
**Geisinger cohort**	0.06 (0.02-0.16)	$5x10^{-08}$	17	0.06 (0.02 - 0.18)	$1x10^{-06}$	17
**UK Biobank**	0.05 (0.02 - 0.1)	$7x10^{-12}$	29	0.08 (0.02 - 0.2)	$1x10^{-06}$	28
**Unrelated Europeans**						
**Probands**	Base		122	Base		110
**Unrelated Family members of proband**	0.5 (0.4 - 0.7)	$1x10^{-04}$	56	1.3 (0.8 - 2.0)	0.3	36
**Unrelated Geisinger cohort**	0.07(0.02 - 0.18)	$3x10^{-07}$	15	0.07(0.02 - 0.19)	$4x10^{-07}$	15
**Unrelated UK Biobank**	0.06(0.02 - 0.1)	$1x10^{-10}$	25	0.09 (0.03 - 0.2)	$6x10^{-06}$	24

Table S7: Univariate and multivariate Cox proportional regression hazard ratios for diabetes for individuals with pathogenic *HNF4A* variants in each unselected cohort relative to *HNF4A*-MODY probands. Hazard ratios (95% CI), p values, and number of all individuals with pathogenic variants (top) and unrelated individuals of European ancestry with pathogenic variants (bottom) in each cohort in univariate (left) and multivariate (right) regression analysis vs. probands (base). *Multivariate regression adjusted for age at recruitment, sex, body mass index, family history of diabetes, and variant type (PTV vs. missense).

	Hazard Ratio (95% CI)	P Value	N	Adjusted Hazard Ratio (95% CI)*	Adjusted P Value*	N
**All individuals**						
**Probands**	Base		37	Base		31
**Family members of proband**	0.2 (0.1 - 0.3)	5.4E-08	43	0.4 (0.2 - 0.9)	0.02	24
**Geisinger cohort**	0.01 (0.002 - 0.1)	2.4E-05	24	0.02 (0.002 - 0.2)	5.9E-04	24
**UK Biobank**	0.004 (0.0006 - 0.04)	2.2E-07	58	0.009 (-0.0008 - 0.09)	6.4E-05	28
**Unrelated Europeans**						
**Probands**	Base		33	Base		29
**Unrelated Family members of proband**	0.3 (0.1 - 0.6)	0.003	15	0.7 (0.3 - 2.0)	0.6	11
**Unrelated Geisinger cohort**	0.02 (0.002 - 0.1)	8.8E-05	19	0.03 (0.003 - 0.3)	2.5E-03	19
**Unrelated UK Biobank**	0.005 (0.0007 - 0.04)	3.9E-07	54	0.009 (0.0009 - 0.1)	7.8E-05	53

Table S8: Univariate and multivariate Cox proportional regression hazard ratios for individuals with pathogenic *HNF4A* p.Arg114Trp in each unselected cohort relative to those of probands. Hazard ratios (95% CI), p values, and number of all individuals with pathogenic variants (top) and unrelated individuals of European ancestry with pathogenic variants (bottom) in univariate (left) and multivariate (right) regression analysis vs. probands (base). *Multivariate regression adjusted for age at recruitment, sex, body mass index, family history of diabetes, and variant type.

	Hazard Ratio (95% CI)	*P* for Heterogeneity
**All *HNF1A***		
**Family members**	11.0 (8.3 - 14.7)	0.005
**Geisinger cohort**	4.0 (2.1 - 7.7)	
**UK Biobank**	15.6 (8.7 - 28.2)	
**Unrelated European *HNF1A***		
**Family members**	7.8 (4.9 - 12.5)	0.002
**Geisinger cohort**	3.8 (1.9 - 7.6)	
**UK Biobank**	18.6 (10.3 - 33.7)	
**All *HNF4A***		
**Family members**	7.6 (4.1 - 14.3)	0.35
**Geisinger cohort**	4.2 (2.1 – 8.4)	
**UK Biobank**	7.7 (4.3 - 13.9)	
**Unrelated European *HNF4A***		
**Family members**	2.8 (1.2 - 6.3)	0.10
**Geisinger cohort**	4.9 (2.4 – 9.8)	
**UK Biobank**	8.7 (4.5 - 17)	
**All *HNF4A* p.Arg114Trp**		
**Family members**	1.3 (0.6 - 2.8)	0.2
**Geisinger cohort**	2.9 (1.7 - 4.9)	
**UK Biobank**	1.7 (0.8 - 3.7)	
**Unrelated European *HNF4A* p.Arg114Trp**		
**Family members**	2.2 (0.6 - 7.8)	0.2
**Geisinger cohort**	4.3 (2.5 - 7.4)	
**UK Biobank**	2.0 (0.9 - 4.5)	

Table S9: Cox proportional hazard ratios for age related onset of diabetes in individuals with and without *HNF1A* or *HNF4A* pathogenic variants in each clinically unselected study cohort. Relative hazard ratios (95% CI) for diagnosis of diabetes in individuals with vs. individuals without *HNF1A*, *HNF4A*, or *HNF4A* p.Arg114Trp variants in each cohort. Cox regression models were applied to all individuals in the cohort or unrelated individuals of European ancestry. Meta-analysis using random-effects models by each condition showed no statistically significant differences in relative hazard ratios for diabetes between cohorts with *HNF4A* but a significant difference with *HNF1A*, although the relative hazard ratios 95% CI between cohorts overlap.

Characteristics	MODY probands (index cases)	MODY family members	Geisinger cohort	UK Biobank
**N**	939	723	32	83
**Age, y**	13.2 (13.2)	35.7 (20.7)	55.4 (18.9)	56.9 (7.9)
**Female Sex, n (%)**	583 (62)	432 (60)	18 (56)	49 (59)
**BMI (kg/m^2)**	23.1 (3.9), n=706	24.7 (4.5), n=290	30.5 (7.4), n=31	27.4 (4.7), n=83
**Diabetes, n (%)**	602 (64)	418 (58)	22 (69)	54 (65)
**Diagnosed with diabetes by baseline biomarkers only, n (%)**	---	---	0)	18 (22)
**Age at diabetes diagnosis, y**	19.2 (11.1)	31.8 (17.1)	49.9 (18.7)	53.0 (9.8)
**Parent with diabetes, n (%)**	689 (73)	362 (50)	12 (38)	34 (41)
**HbA1c, mmol/mol**	46.1 (6.1), n=768	48.4 (8.4), n=331	48.3 (9.9), n=26	47.5 (4.6), n=83
**Fasting glucose, mmol/l**	6.7 (0.8), n=727	6.9 (1.1), n=323	6.9 (1.3), n=22	6.5 (1.1), n=17
**European ancestry, n (%)**	751 (90)	562 (93)	32 (100)	80 (96)
**Unrelated, n (%)**	939 (100)	330 (46)	27 (84)	75 (90)
**PTV, n (%)**	220 (23)	174 (24)	6 (19)	21 (25)

Table S10: Characteristics of *GCK* heterozygotes in MODY probands, proband family members, Geisinger cohort and UK Biobank at recruitment. Values are mean (SD) for continuous variables or number of individuals (%) for categorical variables. The number of individuals with available data is also indicated where appropriate.

	N	Mean (95%CI)	Unadjusted		Adjusted for age		Adjusted for multi-variables*	
			Mean difference vs proband (95% CI)	P vs proband	Mean difference vs proband (95% CI)	P vs proband	Mean difference vs proband (95% CI)	P vs proband
**All individuals**								
**Proband**	768	46.1 (45.7 - 46.6)	base	base	base	base	base	base
**Family members**	331	48.4 (47.5 - 49.3)	2.3 (1.4, 3.2)	4.3E-07	1.5 (0.6, 2.4)	0.002	1.1 (-0.1, 2.2)	0.07
**Geisinger cohort**	26	48.3 (44.3 - 52.3)	2.1 (-0.6, 4.8)	0.1	0.1 (-2.7, 2.9)	1.0	-0.5 (-3.5, 2.5)	0.7
**UK Biobank**	83	47.5 (46.5 - 48.5)	1.3 (-0.2, 2.9)	0.1	-0.6 (-2.4, 1.1)	0.5	-0.9 (-2.8, 1.0)	0.3
**European unrelated**								
**Proband**	630	46.1 (45.6 - 46.6)	base	base	base	base	base	base
**Family members**	131	49.5 (48.2 - 50.8)	3.4 (2.3, 4.6)	2.0E-08	2.6 (1.3, 4.0)	0.0002	1.1 (-0.1, 2.2)	0.07
**Geisinger cohort**	22	46.6 (43.3 - 49.5)	0.3 (-2.4, 3.0)	0.8	-0.9 (-3.8, 1.9)	0.5	-0.5 (-3.5, 2.5)	0.7
**UK Biobank**	74	47.6 (46.6 - 48.7)	1.5 (-0.02, 3.0)	0.05	0.3 (-1.5, 2.1)	0.7	-0.9 (-2.8, 1.0)	0.3

Table S11: Comparison of HbA1c (mmol/mol) in *GCK*-MODY probands vs individuals with *GCK* pathogenic variants in each unselected cohort. Mean (95% CI) and number of all individuals (N, top) and unrelated individuals of European ancestry (bottom) are listed. Univariate, age-adjusted, and multivariate Cox-proportional regression analyses compared HbA1c levels between each cohort and probands (base). The mean difference of HbA1c (95% CI) and p values between each cohort compared to probands is shown. *Adjusted for age at study, sex, and body mass index

	N	Mean (95%CI)	Unadjusted		Adjusted for age		Adjusted for multi-variables*	
			Mean difference vs proband (95% CI)	P vs proband	Mean difference vs proband (95% CI)	P vs proband	Mean difference vs proband (95% CI)	P vs proband
**All individuals**								
**Proband**	727	6.7 (6.7 - 6.8)	base	base	base	base	base	base
**Family members**	323	6.9 (6.8 – 7.0)	0.2 (0.04,0.3)	0.01	0.08 (-0.04,0.2)	0.2	-0.03(-0.2,0.1)	0.7
**Geisinger cohort**	22	6.9 (6.3 - 7.5)	0.2 (-0.2,0.6)	0.3	-0.08 (-0.5,0.3)	0.7	-0.5 (-0.9,-0.02)	0.04
**UK Biobank**	17	6.5 (6 - 7.1)	-0.2 (-0.6,0.2)	0.4	-0.4 (-0.8,0.0)	0.07	-0.6 (-1.1,-0.2)	0.01
**European unrelated**								
**Proband**	601	6.7 (6.7 - 6.8)	base	base	base	base	base	base
**Family members**	106	7.0 (6.8 - 7.2)	0.3 (0.1,0.4)	8.30E-04	0.2 (0.1,0.4)	0.01	0.1 (-0.1,0.4)	0.3
**Geisinger cohort**	18	7.0 (6.4 - 7.6)	0.3 (-0.1,0.6)	0.2	0.2 (-0.2,0.6)	0.3	-0.1 (-0.5,0.4)	0.8
**UK Biobank**	15	6.6 (5.9 - 7.2)	-0.1 (-0.5,0.3)	0.5	-0.2 (-0.6,0.2)	0.4	-0.4 (-0.8,0.1)	0.1

Table S12: Comparison of fasting blood glucose (mmol/l) in *GCK*-MODY probands vs individuals with *GCK* pathogenic variants in each unselected cohort. Mean (95% CI) and number of all individuals (N, top) and unrelated individuals of European ancestry (bottom) are listed. Univariate, age-adjusted, and multivariate Cox-proportional regression analyses compared HbA1c levels between each cohort and probands (base). The mean difference of HbA1c (95% CI) and p values between each cohort compared to probands is shown. *Adjusted for age at study, sex, and body mass index

	% (95%CI)	P vs Probands
**Probands**	97 (96 - 98)	base
**Family members**	96 (94 - 98)	0.7
**Geisinger cohort**	89 (71 - 98)	0.05
**UK Biobank**	96 (90 - 99)	0.5

Table S13: Penetrance of mild hyperglycemia in individuals with *GCK* pathogenic variants in unrelated Europeans. Proportion of individuals with pathogenic *GCK* variants with hyperglycaemia as defined by HbA1c > 39 mmol/mol (5.7%) or fasting blood glucose > 5.6 mmol/L (32.7 mmol/mol). Fisher's exact test p values compared each cohort vs. probands.

		Diabetes	Prediabetes only	Total	OR for prediabetes (95% CI)
**Geisinger**	Individuals without pathogenic variants	31258 (23.6%)	37615 (28.6)	132163	
	HNF1A	9 (64.3%)	1 (7.1%)	14	0.42 (0.01-4.2)
	HNF4A	8 (47%)	2 (11.8%)	17	0.48 (0.05-2.5)
**UK Biobank**	Individuals without pathogenic variants	11477 (5.8%)	54677 (27.5%)	198726	
	HNF1A	11 (50%)	2 (9.1%)	22	0.54 (0.06-2.6)
	HNF4A	11 (38%)	6 (20.7%)	29	1.2 (0.37-3.4)

Table S14. Lack of enrichment for prediabetes in individuals with *HNF1A* and *HNF4A* pathogenic variants in the population cohorts. Number (%percentages) of individuals with and without pathogenic *HNF1A* and *HNF4A* variants with diabetes and prediabetes are indicated. Odds ratios and 95% CI show that there is no increased prevalence for prediabetes in individuals with compared to individuals without pathogenic variants. Diabetes was defined as previously described. Prediabetes was defined as HbA1c > 39 mmol/mol (5.7%) or fasting blood glucose > 5.6 mmol/L (32.7 mmol/mol). Abbreviations: OR odds ratios, CI confidence intervals. See Methods for more details.

Regeneron Genetics Center Banner Author List and Contribution Statements

All authors/contributors are listed in alphabetical order.

RGC Management and Leadership Team
Goncalo Abecasis, Aris Baras, Michael Cantor, Giovanni Coppola, Aris Economides, Luca A. Lotta, John D. Overton, Jeffrey G. Reid, Alan Shuldiner, Katia Karalis and Katherine Siminovitch

Contribution: All authors contributed to securing funding, study design and oversight. All authors reviewed the final version of the manuscript.

Sequencing and Lab Operations
Christina Beechert, Caitlin Forsythe, M.S., Erin D. Fuller, Zhenhua Gu, M.S., Michael Lattari, Alexander Lopez, M.S., John D. Overton, , Thomas D. Schleicher, M.S., Maria Sotiropoulos Padilla, M.S., Louis Widom, Sarah E. Wolf, M.S., Manasi Pradhan, M.S., Kia Manoochehri, Ricardo H. Ulloa.

Contribution: C.B., C.F., A.L., and J.D.O. performed and are responsible for sample genotyping. C.B, C.F., E.D.F., M.L., M.S.P., L.W., S.E.W., A.L., and J.D.O. performed and are responsible for exome sequencing. T.D.S., Z.G., A.L., and J.D.O. conceived and are responsible for laboratory automation. M.S.P., K.M., R.U., and J.D.O are responsible for sample tracking and the library information management system.

Genome Informatics
Xiaodong Bai, , Suganthi Balasubramanian, , Andrew Blumenfeld, Boris Boutkov, , Gisu Eom, Lukas Habegger, , Alicia Hawes, B.S., Shareef Khalid, Olga Krasheninina, M.S., Rouel Lanche, Adam J. Mansfield, B.A., Evan K. Maxwell, Mrunali Nafde, Sean O'Keeffe, M.S., Max Orelus, Razvan Panea, , Tommy Polanco, B.A., Ayesha Rasool, M.S., Jeffrey G. Reid, , William Salerno, , Jeffrey C. Staples,

Contribution: X.B., A.H., O.K., A.M., S.O., R.P., T.P., A.R., W.S. and J.G.R. performed and are responsible for the compute logistics, analysis and infrastructure needed to produce exome and genotype data. G.E., M.O., M.N. and J.G.R. provided compute infrastructure development and operational support. S.B., S.K., and J.G.R. provide variant and gene annotations and their functional interpretation of variants. E.M., J.S., R.L., B.B., A.B., L.H., J.G.R. conceived and are responsible for creating, developing, and deploying analysis platforms and computational methods for analyzing genomic data.

Clinical Informatics:
Michael Cantor, Dadong Li and Deepika Sharma

Contribution: All authors contributed to the clinical informatics of the project

Research Program Management
Marcus B. Jones, Jason Mighty, and Lyndon J. Mitnaul

Contribution: All authors contributed to the management and coordination of all research activities, planning and execution. All authors contributed to the review process for the final version of the manuscript.
